# Comparing the Phenylalanine/Tyrosine Pathway and Related Factors between Keratopathy and No-Keratopathy Groups as Well as between Genders in Alkaptonuria during Nitisinone Treatment

**DOI:** 10.3390/metabo12080772

**Published:** 2022-08-22

**Authors:** Lakshminarayan R. Ranganath, Anna M. Milan, Andrew T. Hughes, Andrew S. Davison, Milad Khedr, Richard Imrich, Mattias Rudebeck, Birgitta Olsson, Brendan P. Norman, George Bou-Gharios, James A. Gallagher

**Affiliations:** 1 Departments of Clinical Biochemistry and Metabolic Medicine, Liverpool University Hospitals NHS Foundation Trusts, Liverpool L7 8XP, UK; 2 William Henry Duncan Building, Department of Musculoskeletal and Ageing Science, Institute of Life Course and Medical Sciences, University of Liverpool, Liverpool L69 3BX, UK; 3 Biomedical Research Centre, Slovak Academy of Sciences, 831 01 Bratislava, Slovakia; 4 OnPoint Science AB, 111 29 Stockholm, Sweden; 5 Garriguella AB, 179 62 Ekerö, Sweden

**Keywords:** sex, keratopathy, alkaptonuria, homogentisic acid, hydroxyphenylpyruvate, hydroxyphenyllactate, phenylalanine, tyrosinaemia

## Abstract

Nitisinone (NIT) causes tyrosinaemia and corneal keratopathy (KP), especially in men. However, the adaptation within the phenylalanine (PHE)/tyrosine (TYR) catabolic pathway during KP is not understood. The objective of this study is to assess potential differences in the PHE/TYR pathway during KP and the influence of gender in NIT-induced tyrosinaemia in alkaptonuria (AKU). Samples of serum and 24 h urine collected from patients treated with NIT during a 4-year randomized study in NIT vs. no-treatment controls (SONIA 2; Suitability Of Nitisinone In Alkaptonuria 2; EudraCT no. 2013-001633-41) at months 3 (V2), 12 (V3), 24 (V4), 36 (V5) and 48 (V6) were included in these analyses. Homogentisic acid (HGA), TYR, PHE, hydroxyphenylpyruvate (HPPA), hydroxyphenyllactate (HPLA) and sNIT were analysed at all time-points in serum and urine in the NIT-group. All statistical analyses were post hoc. Keratopathy occurred in 10 out of 69 AKU patients, eight of them male. Thirty-five sampling points (serum and 24 h urine) were analysed in patients experiencing KP and 272 in those with no-KP (NKP) during NIT therapy. The KP group had a lower HPLA/TYR ratio and a higher TYR/PHE ratio compared with the NKP group (*p* < 0.05 for both). There were 24, 45, 100 and 207 sampling points (serum and 24 h urine) in the NIT group which were pre-NIT female, pre-NIT male, NIT female and NIT male, respectively. The PHE/TYR ratio and the HPLA/TYR ratio were lower in males (*p* < 0.001 and *p* < 0.01, respectively). In the KP group and in the male group during NIT therapy, adaptive responses to minimise TYR formation were impaired compared to NKP group and females, respectively.

## 1. Introduction

Tyrosinaemia ensues when tyrosine cannot be catabolised due to a deficient phenylalanine (PHE)/tyrosine (TYR) pathway. Examples include hereditary tyrosinaemias types 2 (HT-2, OMIM 276600) and 3 (HT-3, OMIM 276710) as well as during nitisinone (NIT) therapy in alkaptonuria (AKU, OMIM 203500) and hereditary tyrosinaemia type 1 (HT-1, OMIM 276700) [1,2,3]. In HT-3 and following NIT therapy the deficiency of 4-hydroxyphenylpyruvate-1,2-dioxygenase (HPPD, EC 1.13.11.27) activity invariably results in tyrosinaemia [3,4].

The PHE/TYR pathway, like other metabolic pathways, is dynamic and capable of adaptation to tyrosinaemia [5]. In steady state in individuals with a normal PHE/TYR pathway, the daily dietary PHE/TYR is completely catabolised and reclaimed by the normal functioning of the PHE/TYR pathway. PHE contributes approximately 60% and TYR 40% to the metabolite flux in the PHE/TYR pathway [6]. PHE, TYR, hydroxyphenylpyruvate (HPPA) and hydroxyphenyllactate (HPLA) are among the main tyrosine pathway metabolites proximal to HPPD inhibition and their measurement can shed light on the state of the pathway before and during NIT therapy. HPPA accumulates during NIT therapy. HPPA can be converted to HPLA by the bidirectional HPPA/HPLA pathway. In addition, HPPA can also reconvert back to TYR due to the bidirectional nature of the tyrosine aminotransferase (TAT). The dominant circulating PHE/TYR pathway metabolite during NIT therapy is TYR which is often an order of magnitude higher than the others; it is therefore possible that TYR may inhibit further conversion of PHE to TYR, through the mechanism of product inhibition, as an adaptive response [5,7].

Circulating and tissue tyrosine concentrations are increased in tyrosinaemia [8]. Undesirable effects of tyrosinaemia include skin rash [9], vitiligo [10], cataract [11], possible cognitive impairment in childhood [12] and corneal dendritiform keratopathy [13], of which the skin rash and corneal keratopathy are reversible if timely tyrosine lowering can be achieved [9,13]. Corneal keratopathy ensued during the development of NIT as an herbicide in rodent toxicology studies [14]. The characterisation of the keratopathy resulted in the knowledge that when circulating TYR was around 900 µmol/L, the corresponding ocular tyrosine concentrations were approximately 3500 µmol/L, at which stage tyrosine crystals formed leading to corneal damage and the keratopathy [15].

AKU is a disorder of the TYR pathway caused by the lack of homogentisate 1,2 dioxygenase (HGD) (EC 1.13.11.5) activity, thereby leading to accumulation of HGA and its consequent damaging effects [16,17]. NIT was approved by the European Medicines Agency as the first disease-modifying therapy for AKU in 2020 [18,19] and decreases HGA [20] by inhibiting HPPD and consequently ameliorates AKU [21,22]. However, inhibition of HPPD also leads to accumulation of metabolites proximal to this inhibition [7], including TYR, leading to severe tyrosinaemia [22].

The development of NIT as a therapy for AKU was carried out in the phase 3 randomised clinical study SONIA 2 (Suitability Of Nitisinone In Alkaptonuria 2) and provided an opportunity to understand whether the maladaptations in the PHE/TYR pathway resulted in those patients experiencing corneal keratopathies [18]. Further, it was noted during the SONIA 2 study that men suffered more keratopathies than women.

Keratopathy was found to occur in 10 of the 69 patients receiving NIT in SONIA 2, even though more than half of all samples had circulating TYR values greater than 900 µmol/L [18]. The question that therefore follows is whether the adaptation of the PHE/TYR pathway during NIT therapy is different in those developing keratopathy. This was one of the hypotheses examined in the present analysis. The keratopathies during NIT therapy in SONIA 2 were mostly in men (Tables S1 and S2) and the reason for this gender difference is not known. The hypothesis that the PHE/TYR pathway could be different in men and women was also studied [18,20,22].

## 2. Materials and Methods

### 2.1. Study Design and Patients

SONIA 2 was a four-year, open-label, evaluator-blinded, multi-centre, randomised, no-treatment controlled, parallel-group study (EudraCT no. 2013-001633-41). The study design is as summarised previously [18]. The study was performed at three investigational sites: Liverpool (UK), Paris (France) and Piešťany (Slovakia). Independent Ethics Committee at each centre approved the study. The aim was to recruit 140 patients aged 25 years or older, with a confirmed diagnosis of AKU and any clinical manifestation in addition to increased HGA: 70 randomised to NIT and 70 to a control (no-NIT) group. All patients provided written informed consent prior to inclusion.

Oral NIT (Orfadin^®^, Swedish Orphan Biovitrum Ltd., Stockholm, Sweden) 10 mg daily was administered in the treated group. The control group did not receive the study drug. There were no restrictions regarding concomitant medications. Patients in both groups could freely use analgesics, anti-inflammatory drugs and others as needed to treat symptoms of AKU. Details of randomisation are as discussed previously [18].

Safety outcomes included the corneal dendritiform keratopathy due to inevitable tyrosinaemia during NIT. The diet of patients in SONIA 2 was not actively managed, apart from providing information sheets regarding eating a lower protein diet. NIT was withdrawn in patients who developed signs of ocular tyrosine-related adverse event. If feasible, once the symptoms of keratopathy had resolved (minimum 2 months after temporary withdrawal), NIT was reintroduced at a lower dose (2 mg daily). Alternatively, the patient was withdrawn from the study. If ocular tyrosine-related symptoms reappeared on the lower 2 mg dose, NIT was permanently withdrawn and the patient was monitored until the symptoms resolved. However, in the present analysis of the SONIA 2 data manuscript, the data collected from these ‘rescued’ patients were used in the data analysis whether the dose of NIT was 10 mg or 2 mg.

### 2.2. Procedures

Although a number of assessments and investigations including collection of medical history and physical examination, comprising of those specific for AKU, have been discussed elsewhere [18], only data relevant to the analyses presented here are shown and discussed further. Patients visited study sites at V1 (Visit 1; baseline), V2 (3 months) and then yearly up to month 48 (V3–V6). Only data from the group randomised to NIT are discussed in the manuscript.

### 2.3. Chemical Analyses

Measurements of NIT, HGA, TYR, PHE, HPPA and HPLA, in serum (indicated as sNIT, sHGA, sTYR, sPHE, sHPPA and sHPLA) and 24 h urine (indicated as uHGA_24_, uTYR_24_, uPHE_24_, uHPPA_24_ and uHPLA_24_) were carried out on all samples collected at the described study visits. Blood samples were collected in plain serum tubes (Sarstedt, Germany). An aliquot of serum was immediately acidified using perchloric acid (10% *v*/*v* 5.8 M), to stabilise the HGA, and kept frozen at −80 °C until analysis. Samples from Paris and Piešťany were transported frozen by courier to Liverpool and all biochemical analyses were performed in the Department of Clinical Biochemistry, Liverpool Clinical Laboratories, Liverpool University Hospital NHS Foundation Trust [18].

The concentrations of sNIT, sHGA, sTYR, sPHE, sHPPA and sHPLA as well as uHGA_24_, uTYR_24_, uPHE_24_, uHPPA_24_ and uHPLA_24_ were measured by liquid chromatography tandem mass spectrometry using previously published methods [7,23]. TYR and PHE and their metabolites were quantitated and multiplied by the 24 h urine volumes to yield daily metabolite excretion (µmol/day) before and during treatment with nitisinone.

At each visit, 24 h urine was collected for the measurement of urea, creatinine and PHE/TYR pathway metabolites into 2.5 L bottles containing 30 mL of 5N H_2_SO_4_ and stored away from direct sunlight. The weight of the collected urine was recorded and used as the volume in the calculations of amount of urea excreted assuming a density of 1 g/mL. An aliquot of the collected urine was frozen and kept at −80 °C until analysis.

Urine urea and creatinine were photometrically assayed in the 24 h urine collection on a Roche Cobas 701 using an automated assay (hydrolysis with urease and subsequent oxidation of NADH). Urine urea was used to objectively estimate dietary protein intake in keeping with other studies [24,25]. Urine creatinine was measured using a validated Jaffe reaction. All methods were performed in accordance with the relevant guidelines and regulations.

### 2.4. Statistical Analysis

All statistical analyses were post hoc. Continuous variables are presented using mean and standard deviation (SD). Analyses were performed using Graphpad InStat 3 software (version number 3.1, California, USA); *p*-values <0.05 were considered statistically significant. Sampling points (for serum and 24 h urine) were classified into keratopathy (KP) and no-keratopathy (NKP) for analysis by ANOVA. Similarly, influence of gender was analysed as female pre-NIT, male pre-NIT, female NIT and male NIT, also by ANOVA, for the relevant sampling points (Tukey-Kramer for multiple comparisons).

## 3. Results

### 3.1. Demographics

In the SONIA 2, 69 patients were randomly assigned to receive NIT 10 mg daily. Of these, 55 completed the study. The main reason for discontinuation was adverse event corneal keratopathy (n = 10) [18]. Demographic data and baseline characteristics of the sampling points from the 69 NIT-treated patients are shown in Table 1. There were 376 out of a possible 414 sampling points where blood and 24 h urine samples were collected in the NIT-group between visits 1 (V1) and 6 (V6). The missing sampling points were those of patients withdrawing from study and due to poor compliance (absent or very low NIT in serum).

### 3.2. Keratopathy (KP) and No-Keratopathy (NKP) Demographics

Ten patients experienced KP, with nine confirmed by slit-lamp examination, consisting of two women and eight men (Table S1); one female patient had clinical features of KP unconfirmed by slit-lamp examination due to not returning for this assessment. Of the patients with slit-lamp confirmed KP, resolution following discontinuation was only confirmed in eight patients, due to one patient not returning for this assessment. The onset of KP varied from one to 36 months. Both unilateral and bilateral KPs were noted and sTYR ranged from 609–1236 µmol/L. Eight patients were switched to NIT 2 mg with four experiencing KP on this dose. Four patients were still on NIT 2 mg at end of the study, the other six having discontinued participation.

There were 307 sampling points between V2 and V6 available for comparison of KP and NKP groups (Table 1). The mean (SD) age of 35 sampling points between V2 and V6 in the 10 patients in the NIT KP group was 44.6 (10.4) years. Similarly, the mean (SD) age of 272 sampling points between V2 and V6 in the 59 patients in the NIT NKP group was 51.4 (10.9) years. Data from V1 visits were not used in the KP and NKP comparisons.

### 3.3. Female and Male Demographics

A higher proportion of subjects were male (n = 45, 65%) compared to female (n = 24, 35%). The male group was significantly younger with a mean (SD) age of 47.4 (11.9) years compared to the female group with mean (SD) values of 51.9 (9.6) years (*p* < 0.001).

### 3.4. Comparison of Metabolite and Other Analytes in the KP and NKP Groups

**Figure 1:**
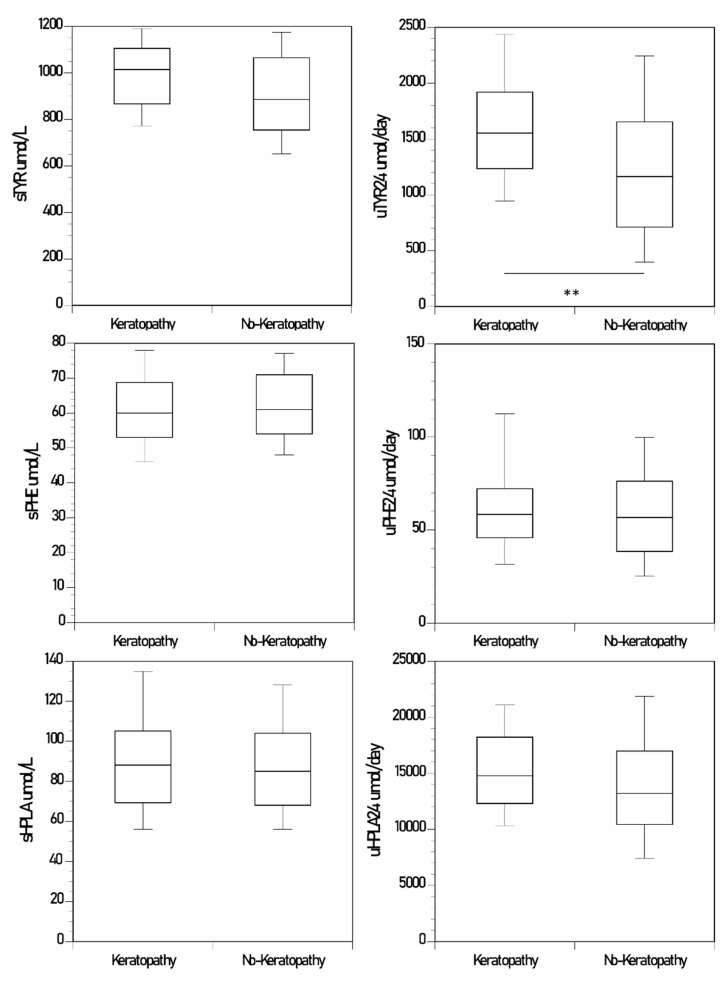
Changes in sTYR and uTYR_24_, sPHE and uPHE_24_, and sHPLA and uHPLA_24_ in the nitisinone group of the SONIA 2. (*p*-values indicated for comparison between keratopathy and no-keratopathy sampling points where statistical significance was achieved; keratopathy sampling points n = 35; no-keratopathy sampling points n = 272) (data shown as boxplots with median) (statistical significance *p* expressed * < 0.05, ** < 0.01, and *** < 0.001 respectively).

**Figure 2:**
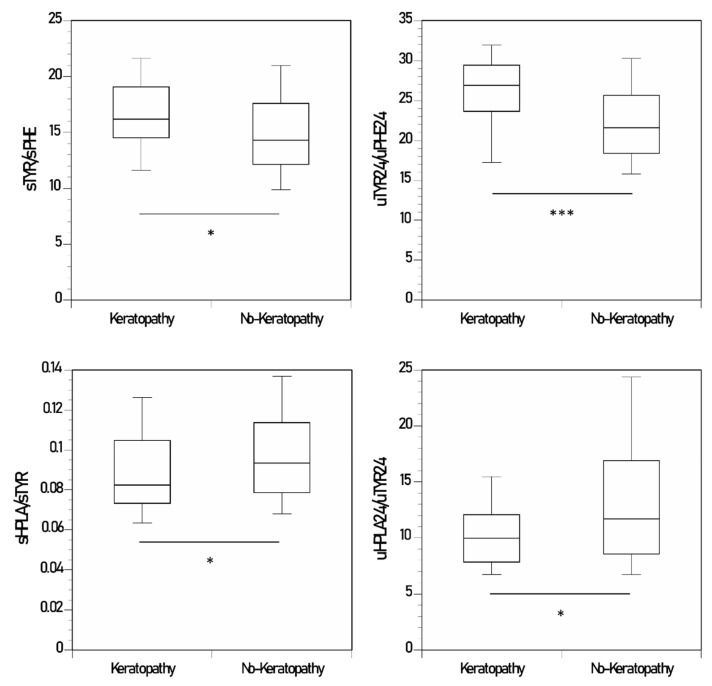
Changes in sTYR/sPHE & uTYR_24_/uPHE_24_, and sHPLA/sTYR & uHPLA_24_/uTYR_24_ in the nitisinone group of the SONIA 2. (*p*-values indicated for comparison between keratopathy and no-keratopathy sampling points where statistical significance was achieved; keratopathy sampling points n = 35; no-keratopathy sampling points n = 272 (data shown as boxplots with median) (statistical significance *p* expressed * < 0.05, ** < 0.01 and *** < 0.001, respectively).

Data are shown as mean (SD) and in boxplots in figures as medians (Table 1 and Figure 1, Figure 2 and Figures S1–S3). uCREAT_24_ was significantly higher in KP (13.1 (7.8)) than in NKP (10.5 (6.6)) (*p* < 0.05) (Table 1, Figure S1). uUREA_24_ tended to be higher in KP (326 (97)) than in NKP (290 (148)) (*p* = 0.16) (Table 1, Figure S1). KP patients were significantly younger (44.6 (10.4)) than the NKP (51.4 (10.9)) (*p* < 0.0001) (Table 1, Figure S1). sHGA was significantly higher in KP (1.9 (2.1)) than in NKP (0.7 (1.1)) (*p* < 0.0001) (Table 1, Figure S2). sTYR tended to be higher in KP in serum (*p* = 0.10) and significantly higher in urine (*p* < 0.01) (Table 1, Figure 1). sPHE and uPHE_24_ tended to be slightly lower in KP, but did not achieve statistical significance (Table 1, Figure 1). HPPA and HPLA tended to be higher in KP in serum and in urine but did not achieve statistical significance (Table 1, Figure 1 and Figure S2).

### 3.5. Keratopathy and No-Keratopathy Comparison of Ratio of Metabolites

Data are shown as mean (SD) (Table 1 and Figure 1, Figure 2 and Figures S1–S3). sTYR/sPHE and uTYR_24_/uPHE_24_ were significantly higher in KP (16.6 (4.0) and 25.9 (5.8), respectively) than NKP (14.8 (4.1) and 22.2 (5.7), respectively) (*p* < 0.05 and < 0.001, respectively) (Table 1, Figure 2). sHPPA/sTYR and uHPPA_24_/uTYR_24_ were significantly lower in KP (0.04 (0.009) and 11.3 (3.2), respectively) than NKP (0.05 (0.02) and 15.8 (9.5), respectively) (*p* < 0.07 and <0.001 respectively) (Table 1, Figure S3). sHPLA/sTYR and uHPLA_24_/uTYR_24_ were significantly lower in KP (0.09 (0.02) and 10.8 (4.8), respectively) than NKP (0.10 (0.03) and 14.6 (10.9), respectively) (*p* < 0.05 and < 0.05, respectively) (Table 1, Figure 2).

### 3.6. Comparison of Metabolite and Other Analytes in Males and Females

**Figure 3:**
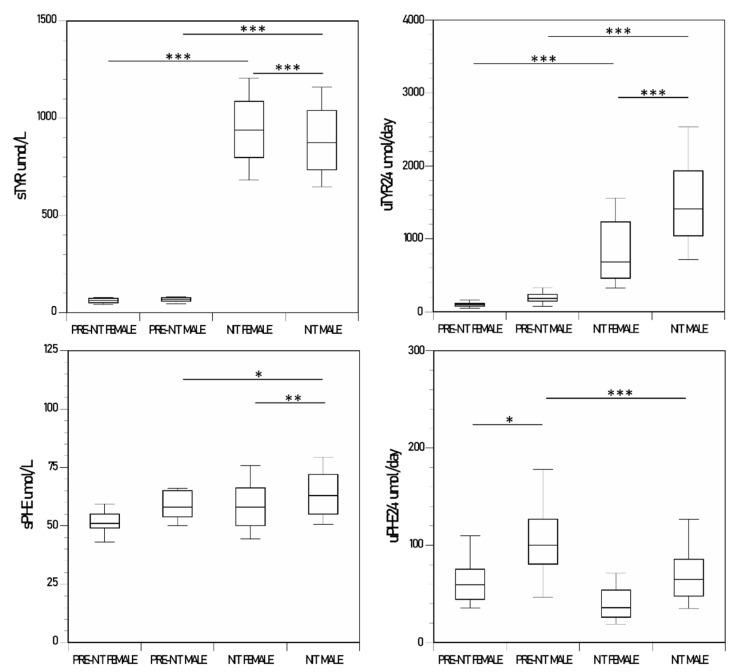
Changes in sTYR and uTYR_24_ and sPHE and uPHE_24_, in the nitisinone group of the SONIA 2. (*p*-values indicated for comparison between female pre-nitisinone (n = 24), male pre-nitisinone (n = 45), female nitisinone (n = 120), male pre-nitisinone (n = 225), sampling points where statistical significance was achieved) (data shown as boxplots with median) (statistical significance *p* expressed * < 0.05, ** < 0.01 and *** < 0.001, respectively).

**Figure 4:**
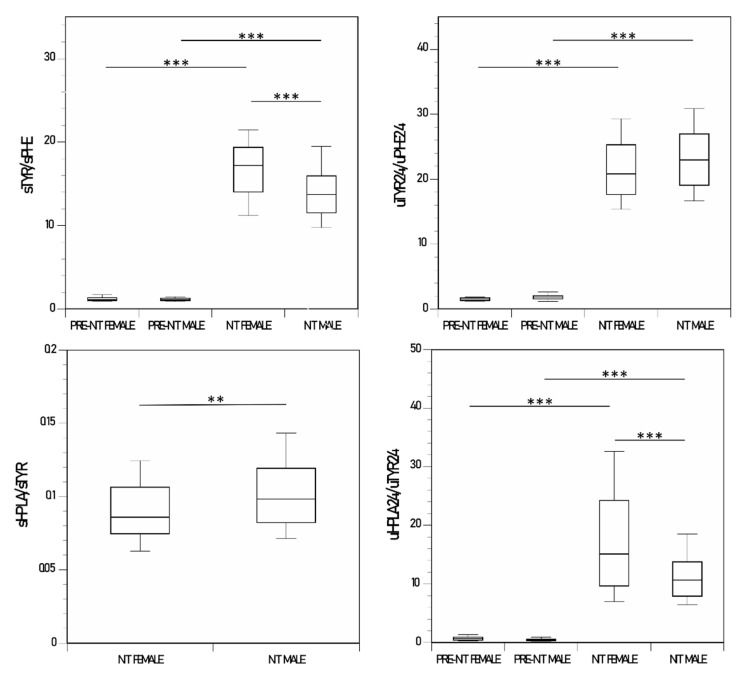
Changes in sTYR/sPHE and uTYR_24_/uPHE_24_, and sHPLA/sTYR and uHPLA_24_/uTYR_24_, in the nitisinone group of the SONIA 2. (*p*-values indicated for comparison between female pre-nitisinone (n = 24), male pre-nitisinone (n = 45), female nitisinone (n = 120), male pre-nitisinone (n = 225), sampling points where statistical significance was achieved) (data shown as boxplots with median) (statistical significance *p* expressed * < 0.05, ** < 0.01 and *** < 0.001, respectively).

Data are shown as mean (SD) (Table 1 and Figure 3, Figure 4 and Figures S4–S6). uCREAT_24_ was higher in the male group both before and during NIT therapy, reaching statistical significance only during NIT therapy (*p* < 0.001). There was no difference in uCREAT_24_ between pre-NIT and NIT groups in either males or females. uUREA_24_ was higher in the male group both before and during NIT therapy, reaching statistical significance only during NIT therapy (*p* < 0.001). There was no difference in uUREA_24_ between pre-NIT and NIT groups in either males or females; however, females had lower uUREA_24_/kg body weight during NIT therapy (*p* < 0.05). The male group was younger than the female group both before and during NIT therapy, reaching statistical significance only during NIT therapy (*p* < 0.01). The male group was significantly heavier than the female group both pre-NIT and during NIT (*p* < 0.01) (Table 1, Figure S4).

Both the male and female groups showed a significant decrease in sHGA and uHGA_24_ after NIT therapy (Figure S5). Both the sHGA and uHGA_24_ were significantly higher in the male group pre-NIT (*p* < 0.05 and < 0.001, respectively) (Table 1, Figure S5).

Both the male and female groups showed a significant increase in sTYR and uTYR_24_ after NIT therapy (Figure 3). sTYR was higher in the female group during NIT therapy (*p* < 0.001), whereas uTYR_24_ was higher in the male group during NIT therapy (*p* < 0.001) (Table 1, Figure 3).

The sPHE was higher in the male group during NIT therapy compared to values prior to NIT therapy (*p* < 0.01), with a similar trend in the female group. The uPHE_24_ was higher in the male group prior to NIT therapy compared to values during NIT therapy (*p* < 0.01), with a similar trend in the female group. The sPHE and the uPHE_24_ were higher in the male group compared to the female group, reaching statistical significance only during NIT therapy (*p* < 0.01 and < 0.05 respectively) (Table 1, Figure 3).

The sHPPA and sHPLA were below the lower limit of quantification (LLoQ) prior to NIT therapy. The uHPPA_24_ and uHPLA_24_ increased significantly from low levels pre-NIT to extremely high levels during NIT therapy both in the male and female groups men (*p* < 0.01 and < 0.001, respectively, for uHPPA_24_, and *p* < 0.001 and < 0.001, respectively, for uHPLA_24_). The uHPPA_24_ and uHPLA_24_ were higher in the male group than the female group during NIT therapy (*p* < 0.001 for both) (Table 1 and Figure S5).

### 3.7. Female and Male Comparison of Ratio of Metabolites

The sTYR/sPHE and the uTYR_24_/uPHE_24_ ratios were higher during NIT therapy than before starting NIT in the male and female groups (*p* < 0.001 for both). In addition, the sTYR/sPHE ratio was higher in the female group than in the male group during NIT therapy (*p* < 0.001) (Table 1, Figure 4).

sHPPA/sTYR ratio was not available pre-NIT as the HPPA was below the LLoQ; however, during NIT therapy there was a trend towards a higher sHPPA/sTYR ratio in the male group compared with the female group. The uHPPA_24_/uTYR_24_ ratio was significantly higher during NIT therapy than prior to NIT therapy in both the male and female groups (*p* < 0.001 for both). The uHPPA_24_/uTYR_24_ ratio was significantly higher in the female group than in the male group during NIT therapy (*p* < 0.001) (Table 1, Figure S6).

sHPLA/sTYR ratio was not available pre-NIT as the HPLA was below the LLoQ; however, during NIT therapy the sHPLA/sTYR ratio was higher in the male group than in the female group. The uHPLA_24_/uTYR_24_ ratio was significantly higher during NIT than prior to NIT therapy in both the male and female groups (*p* < 0.001 for both). The uHPLA_24_/uTYR_24_ ratio was significantly lower in the male group than in the female group during NIT therapy (*p* < 0.001) (Table 1, Figure 4).

## 4. Discussion

sTYR from the 307 samples, 35 from KP and 272 from NKP, from NIT-treated AKU patients, showed a median value of approximately 900 µmol/L with 50.3% of the values greater than 900 µmol/L. Surprisingly, most of the sampling points were not associated with keratopathy despite sTYR levels greater than 900 µmol/L The 900 µmol/L threshold was used for the development of keratopathy because sTYR shows a known relationship with the ocular TYR, and management of nitisinone therapy is geared to maintaining sTYR well below 900 µmol/L [15,26,27]. HGA, HPPA and HPLA values are below the LLoQ without NIT, and sNIT was used as a measure of HPPD inhibition. The metabolites of the PHE/TYR pathway themselves could influence each other and determine the circulating concentration of these metabolites including sTYR as well as the development of keratopathy.

Since dietary PHE and TYR intake reflected in daily protein intake analysed as uUREA_24_ affect metabolite accumulation in NIT-induced HPPD inhibition, this was examined in the KP and NKP groups and not found to be significantly different, suggesting that the differences between KP and NKP were not mainly due to differences in dietary PHE and TYR intakes. Despite the KP sample group being younger, the expected decline in protein intake with age was not observed compared to the NKP group [28,29,30]. On the other hand, most of the KP patients were male and during NIT therapy females showed decrease in uUREA_24_/kg, but not males.

The sHGA and uHGA_24_ were significantly different between the KP and NKP groups, with values being higher in KP group; this is consistent with sNIT being lower in the KP group since the nitisinone dose was decreased from 10 mg to 2 mg daily once keratopathy developed. Despite this decrease in sNIT as well as the NIT dose in the KP group, sTYR tended to be higher, while sPHE, sHPPA and sHPLA were not significantly lower. The uTYR_24_ was higher in the KP group and consistent with greater TYR filtered by the kidney and increased proportional loss of TYR in the urine. Despite the lower sNIT and lower NIT dose, the uPHE_24_, uHPPA_24_ and uHPLA_24_ were not significantly lower. These metabolic features of KP compared to NKP groups, despite the lower sNIT and NIT dose, are consistent with a predisposition to higher TYR in the KP group.

The examination of the metabolite ratios was more informative in terms of explaining the higher frequency of keratopathy in the KP group. Increased HPPA resulting from NIT-induced HPPD inhibition could lead to increased flux down the HPLA pathway, a beneficial effect as it minimises conversion of HPPA back to TYR. It is therefore noteworthy that a significantly lower HPLA/TYR ratio, both in the serum and urine, was seen in the KP group consistent with relatively decreased HPLA formation compared to TYR. The lower HPPA/TYR ratio in urine with a similar trend in the serum in KP group also supports increased tyrosine generation (Figure 2 and Figure S3).

Tyrosinaemia could inhibit the conversion of PHE to TYR and provide a further adaptive mechanism to minimise KP formation. Since the usual proportion of PHE and TYR in the human diet is 60:40 [6], PHE contributes significantly to the flux down the pathway in steady state, and adaptation of PHE catabolism during tyrosinaemia could be quantitatively significant. When the relative concentrations of PHE and TYR were compared in the KP and NKP groups, the TYR/PHE ratio, in both the serum and urine, was higher in the KP than in the NKP group. This suggests that there was a lesser inhibition of conversion of PHE to TYR in the KP group, consistent with the development of keratopathy in this group.

**Figure 5:**
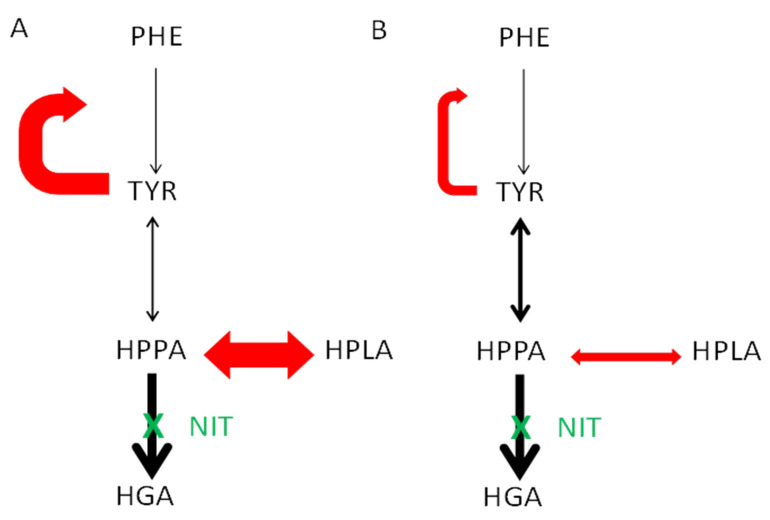
(**A**,**B**) A cartoon representation of changing relationships between PHE, TYR, HPPA and HPLA during NIT therapy. In A, the figure highlights the state of the pathway in NKP group, showing lesser conversion of PHE to TYR (solid black arrow) inhibited more by TYR (more prominent curved red arrow), and similarly more conversion of HPPA to HPLA (a straight solid red arrow) as well as lesser conversion from HPPA to TYR (a straight black arrow). In B, the figure highlights the state of the pathway in the KP group, showing greater conversion of PHE to TYR (thin solid black arrow) inhibited less by TYR (less prominent curved red arrow), as well as lesser conversion of HPPA to HPLA (a straight solid red arrow) and more conversion from HPPA to TYR (a straight black arrow).

In the current dataset from the SONIA 2 NIT-treated cohort, KP was more frequent in males; an examination of published cases of keratopathy during NIT use in AKU revealed that 12 out of the 14 cases were males. In the dataset comparing female and male groups, uUREA_24_ was higher in the male group both before and during NIT, which is consistent with greater daily protein intake in males [28,29]. It is necessary to remember that usual diet may be different from day to day and from that on the sampling day. The uUREA_24_/kg body weight was higher in males during NIT, suggesting that compliance with advice to decrease protein intake during NIT was better in females. Males on NIT therapy were younger than females on NIT therapy and this is due to females having delayed onset of disease [30,31]. Males were heavier than females pre-and during NIT therapy, and had more muscle mass as evidenced by uCREAT_24_ during NIT therapy. These data demonstrate that men have to adapt to a greater daily PHE/TYR load during NIT therapy (Figure 5 and Figure S7).

Both the male and female groups showed the expected decrease in sHGA and uHGA_24_ after NIT therapy. However, the sHGA and uHGA_24_ were higher in the male group pre-NIT, which is consistent with higher daily protein intakes, despite which the sTYR was higher in females during NIT therapy for reasons which are not clear. The higher sNIT in females may at least partly account for the trend to higher sTYR. The lower total body water in females is less than in males and may partly account for the higher sNIT in females due to the fixed dosing of nitisinone irrespective of lower mean body weight and therefore a smaller volume of distribution for NIT. The lower total body water in women results in a smaller volume of distribution for the accumulating TYR. In addition, the uTYR_24_ in females was lower than in males during NIT therapy and suggests greater reabsorption of TYR [32], perhaps due to greater innate reabsorption efficiency in females or due to greater compliance with advice to reduce protein intake during NIT therapy. Males in the SONIA 2 had more KP despite females having higher sTYR and the reason for this difference remains obscure.

It has been suggested that conversion of PHE to TYR may be greater in the female sex, by up to 20%, and may also form part of the explanation for the higher sTYR in the female group in the present analysis [33]. Males may also be associated with higher activity of PHE/TYR pathway, with greater dietary intakes, and with higher concentrations of these amino acids compared to females [34]. The sPHE was higher in males during NIT therapy compared to values prior to NIT therapy, with a similar trend in women; this finding is consistent with our recent publication showing that adaptations to tyrosinaemia include minimising further conversion of PHE to TYR, but also due to greater protein intake in males [5]. Further, the fact that the increase in sPHE during NIT is more notable in males is consistent with the fact that males have a lower phenylalanine hydroxylase activity than females; this mechanism may also serve to explain the higher sPHE in males than in females during NIT therapy [35]. The higher uPHE24 in the males prior to NIT therapy compared to values during NIT therapy, with a similar trend in females, may reflect an adaptive response in the kidney for more efficient reabsorption due to decreasing PHE availability consequent to compliance with protein restriction advice during NIT therapy. The uPHE24 was lower in females than males, especially prior to NIT therapy, which may be due to more efficient renal reabsorption in females, similar to the renal handling of TYR, as discussed earlier [32]. The uHPPA24 and uHPLA24, urine being the major route of organic anion elimination, increased markedly during NIT therapy both in males and females, as described previously [7,26]. The higher uHPPA24 and uHPLA24 in males compared to females during NIT therapy is consistent with a larger daily PHE/TYR load in males due to greater protein intake.

PHE/TYR pathway metabolite ratios are less dependent on protein intake and may better reflect differences in pathway adaptation between males and females during NIT therapy. The uHGA24/uTYR24 ratio was higher in females than in males prior to NIT therapy and may reflect differences in renal handling of TYR as discussed earlier. The lower sTYR/sPHE ratio in males than in females during NIT therapy could be attributed to lower sTYR and/or the higher sPHE in males during NIT therapy. If basal PHE conversion to TYR is lower in males as suggested, then it could be argued that there is less room to adapt further by decreasing PHE to TYR conversion in males relative to females, a potential predisposition to higher TYR and keratopathy. The sHPLA/sTYR ratio was higher in males than in females and may reflect the higher sTYR in females with similar sHPLA in males and females. The lower uHPLA24/uTYR24 ratio in males than in females during NIT therapy may be due to relative deficiency of HPLA formation compared to TYR, and in keeping with the increased prevalence of KP in males; uHPLA24 may be considered more reliable as an indicator of the HPLA pathway given their abundance in the urine compared to the serum. However, more data analysis is needed as the changes in sHPLA/sTYR and the uHPLA24/uTYR24 ratios are diametrically opposite in males and females during NIT therapy.

Our data in SONIA 2 comparing males and females show a non-statistical 8.4% sTYR increase in females during NIT therapy, greater reabsorption of tyrosine in the kidney, smaller volume of distribution (lower weight and likely lower total body water due to higher fat content) and higher sNIT, suggesting that the female group should have been more likely to develop KP. This is balanced by the fact that uUREA_24_ decreased from pre-NIT to during NIT in the female group by 15.9% compared to the 2.7% change in the male group; similarly, uUREA_24_/kg decreased from pre-NIT to during NIT in the female group by 20.9% compared to the 4.8% change in the male group. The change in protein intake, reflected by the uUREA_24_ and uUREA_24_/kg in the female group, may have mitigated the higher KP risk in this group. However, one cannot exclude additional factors to explain this conundrum.

There are limitations in the current analyses. We had to rely on analysing all serum and urine sample points to generate a large enough number to provide a worthwhile analysis in view of the rarity of AKU. This is even more of an issue for the KP/NKP comparisons as there were only a small proportion of patients who developed KP and in whom the full 4-year sampling was lacking. As part of the KP rescue plan, the dose of NIT had to be reduced, which impacts the data, even though half the patients switched to the lower 2 mg dose developed KP. The annual sampling points do not reflect fluctuations in daily dietetic intake, including protein. The proportion of pre-NIT samples when comparing males and females are much smaller than the samples collected during NIT.

## 5. Conclusions

Analysis of our SONIA 2 data in NIT-treated patients revealed that 10 patients developed KP, eight of whom were males. Severe tyrosinaemia was highly prevalent during NIT therapy in SONIA 2. The inhibition of the conversion of PHE to TYR was lower in the KP group and this insufficient adaptive response to minimise TYR formation may predispose to KP. The KP group also had a lower HPLA/TYR ratio consistent with relatively decreased HPLA formation allowing more conversion of HPPA to TYR increasing the likelihood of tyrosinaemia-induced KP. In males there was lesser inhibition of the PHE to TYR conversion during NIT therapy, predisposing males to keratopathy. The uHPLA_24_/uTYR_24_ ratio was lower in males during NIT therapy, which is suggestive of a relative deficiency of HPLA formation, and in keeping with the increased prevalence of KP in males. However, more data analyses are required to understand the differences in males and females with respect to the development of KP.

## Figures and Tables

**Table 1:** Age, weight, uUREA_24_, uUREA_24_/kg, uCREAT_24_ and measured metabolic data in NIT-treated AKU patients based on keratopathy status and sex.

Age, Weight, uUREA_24_, uUREA/kg, uCREAT_24_ and Measured Metabolic Data in NIT-Treated AKU Patients Based on Keratopathy Status and Sex						
	Keratopathy Status		Sex			
	Keratopathy (n = 35)	No-Keratopathy (n = 272)	Female		Male	
			Pre-NIT (n = 24)	NIT (n = 100)	Pre-NIT (n = 45)	NIT (n = 207)
Age years	44.6 (10.4) ***	51.4 (10.9)	51.9 (9.6)	54.0 (9.5)	47.4 (11.9)	49.5 (11.8)
Weight Kg	75.9 (7.1)	78.1 (16.3)	66.3 (15.1)	70.1 (16.3)	79.2 (12.6)	81.5 (13.5)
uUREA_24_ mmol/day	326 (97)	290 (148)	277 (95)	233 (102)	333 (88)	324 (148)
uUREA mmol/Kg	4.3 (1.3)	3.8 (1.9)	4.3 (1.5)	3.4 (1.6)	4.2 (1.0)	4.0 (1.8)
uCREAT_24_ mmol/day	13.1 (7.8) *	10.5 (6.6)	8.2 (2.3)	7.5 (3.1)	11.4 (2.7)	12.4 (7.3)
sHGA µmol/L	1.9 (2.1) ****	0.7 (1.1)	27.9 (10.4)	0.77 (1.5)	31.7 (11.2)	1.1 (2.6)
sTYR µmol/L	982 (167)	913 (231)	62 (17)	968 (256)	67 (14)	893 (214)
sPHE µmol/L	61.4 (13.3)	63.4 (14)	52.1 (7.3)	59.4 (15)	59.3 (9.6)	64.9 (13)
sHPPA µmol/L L	36.6 (6.9)	39.6 (18.7)	-	39.4 (27)	-	39.3 (12.3)
sHPLA µmol/L	88.3 (29.9)	90.9 (32.6)	-	86.1 (38.3)	-	89.6 (33.2)
sNIT µmol/L	3.7 (2.7) **	5.1 (2.7)	-	5.5 (3.3)	-	4.4 (2.4)
uHGA_24_ µmol /day	645 (882) ****	202 (481)	31,024 (13447)	158 (521)	37,098 (12575)	298 (571)
uTYR_24_ µmol /day	1672 (858) **	1290 (765)	100 (46)	867 (491)	196 (88)	1555 (801)
uPHE_24_ µmol /day	68.6 (41.7)	61.5 (38.7)	65 (28)	41 (21)	152 (332)	73 (41)
uHPPA_24_ µmol /day	17,566 (6494)	16,375 (8406)	75 (39)	13,497 (5719)	189 (152)	17,939 (8813)
uHPLA_24_ µmol /day	15,987 (6300)	14211 (6081)	63 (28)	11,942 (4501)	99 (76)	15,585 (6441)
sHGA/sTYR	0.002 (0.003) ****	0.001 (0.001)	0.46 (0.16)	0.0008 (0.001)	0.49 (0.18)	0.003 (0.03)
sTYR/sPHE	16.6 (4.0) *	14.8 (4.1)	1.2 (0.3)	16.8 (4.2)	1.1 (0.24)	14.1 (3.9)
sHPPA/sTYR	0.04 (0.009)	0.05 (0.02)	-	0.04 (0.03)	-	0.045 (0.01)
sHPPA/sHPLA	0.46 (0.17)	0.47 (0.26)	-	0.49 (0.37)	-	0.46 (0.16)
sHPLA/sTYR	0.09 (0.02) *	0.10 (0.03)	-	0.09 (0.03)	-	0.1 (0.03)
uHGA_24_/uTYR_24_	0.43 (0.61) *	0.18 (0.63)	354 (157)	0.22 (0.92)	219 (107)	0.21 (0.44)
uTYR_24_/uPHE_24_	25.9 (5.8) ***	22.2 (5.7)	1.53 (0.3)	21.6 (5.9)	1.9 (0.8)	23.2 (5.8)
uHPPA_24_/uTYR_24_	11.3 (3.2) **	15.8 (9.5)	0.85 (0.12)	19.8 (11.7)	0.85 (0.56)	13.1 (6.6)
uHPPA_24_/uHPLA_24_	1.1 (0.32)	1.2 (0.93)	1.52 (1.0)	1.3 (1.4)	1.66 (1.3)	1.18 (0.4)
uHPLA_24_/uTYR_24_	10.8 (4.8) *	14.6 (10.9)	0.74 (0.4)	18.5 (13.6)	0.49 (0.27)	12.1 (7.7)

Variation among column means is significantly greater than expected by chance with *p* <: * < 0.05; ** < 0.01; *** < 0.001; **** < 0.0001; keratopathy and no-keratopathy comparisons are shown in columns 2 and 3; S—serum; uX_24_—24 h urine; HGA—homogentisic acid; TYR—tyrosine; PHE—phenylalanine; HPPA—4-hydroxyphenylpyruvate; HPLA—4-hydroxyphenyllactate; NIT—nitisinone; CREAT—creatinine.

## Data Availability

The data presented in this study are available in the main article and the Supplementary Materials.

## Supplementary Materials

The following supporting information can be downloaded at: https://www.mdpi.com/article/10.3390/metabo12080772/s1, Figure S1. Changes in uCREAT_24_, uUREA_24_, age and body weight in nitisinone group of SONIA 2, Figure S2. Changes in sHGA & uHGA_24_, and sHPPA & uHPPA_24_ in the nitisinone group of the SONIA 2, Figure S3. Changes in sHGA/sTYR & uHGA_24_/uTYR_24_, sHPPA/sTYR & uHPPA_24_/uTYR_24_, and sHPPA/sHPLA & uHPPA_24_/uHPLA_24_ in the nitisinone group of the SONIA 2, Figure S4. Changes in uCREAT_24_, uUREA_24_, age and body weight in the male and female subgroups of the nitisinone group of the SONIA 2, Figure S5. Changes in sHGA & uHGA_24_, sHPPA & uHPPA_24_, and sHPLA & uHPLA_24_ in the male and female subgroups of the nitisinone group of the SONIA 2, Figure S6. Changes in sHGA/sTYR & uHGA_24_/uTYR_24_, sHPPA/sTYR & uHPPA_24_/uTYR_24_, and sHPPA/sHPLA & uHPPA_24_/uHPLA_24_ in the male and female subgroups of the nitisinone group of the SONIA 2, Figure S7. The phenylalanine/tyrosine pathway. The PHE/TYR metabolic pathway highlights the site of the enzyme defect observed in AKU and the site of action of nitisinone, a reversible competitive inhibitor of 4-hydroxyphenylpyruvate dioxygenase. The pathway also illustrates the dynamic relationships between HPPA, TYR and HPLA, key interactions after introduction of nitisinone; Table S1. Details of keratopathy in SONIA 2 and related nitisinone studies in AKU, Table S2. Dates of start and end of 10 mg and 2 mg in patients developing keratopathy.
