# Supplementary material for: Comparing the Phenylalanine/Tyrosine Pathway and Related Factors between Keratopathy and No-Keratopathy Groups as Well as between Genders in Alkaptonuria during Nitisinone Treatment
Source: Metabolites. 2022 Aug 22;12(8):772. doi: 10.3390/metabo12080772 (PMC9416442; doi:10.3390/metabo12080772)
Supplement: Supplementary file 1 [file metabolites-12-00772-s001.zip › metabolites-1826743-supplementary.pdf]

Supplementary data

Legend to supplementary tables and figures

Supplementary table

Table S1. Details of keratopathy in SONIA 2 and related nitisinone studies in AKU

Table S2. Dates of start and end of 10mg and 2mg in patients developing keratopathy

Supplementary figures

Figure S1. Changes in $uCREAT_{24}$, $uUREA_{24}$, age and body weight in nitisinone group of SONIA 2. (p values indicated for comparison between keratopathy and no-keratopathy sampling points where statistical significance was achieved; keratopathy sampling points n = 35; no-keratopathy sampling points n = 272) (data shown as boxplots with median) (statistical significance p expressed *<0.05, **<0.01, and ***<0.001 respectively).

Figure S2. Changes in sHGA & $uHGA_{24}$, and sHPPA & $uHPPA_{24}$ in the nitisinone group of the SONIA 2. (p values indicated for comparison between keratopathy and no-keratopathy sampling points where statistical significance was achieved; keratopathy sampling points n = 35; no-keratopathy sampling points n = 272) (data shown as boxplots with median) (statistical significance p expressed *<0.05, **<0.01, and ***<0.001 respectively).

Figure S3. Changes in sHGA/sTYR & $uHGA_{24}/uTYR_{24}$, sHPPA/sTYR & $uHPPA_{24}/uTYR_{24}$, and sHPPA/sHPLA & $uHPPA_{24}/uHPLA_{24}$ in the nitisinone group of the SONIA 2. (p values indicated for comparison between keratopathy and no-keratopathy sampling points where statistical significance was achieved; keratopathy sampling points n = 35; no-keratopathy sampling points n = 272) (data shown as boxplots with median) (statistical significance p expressed *<0.05, **<0.01, and ***<0.001 respectively).

Figure S4. Changes in $uCREAT_{24}$, $uUREA_{24}$, age and body weight in the male and female subgroups of the nitisinone group of the SONIA 2. (p values indicated for comparison between female pre-nitisinone [n = 24], male pre-nitisinone [n = 45], female nitisinone [n = 120], male pre-nitisinone [n = 225], sampling points where statistical significance was achieved) (data shown as boxplots with median) (statistical significance p expressed *<0.05, **<0.01, and ***<0.001 respectively).

Figure S5. Changes in sHGA & $uHGA_{24}$, sHPPA & $uHPPA_{24}$, and sHPLA & $uHPLA_{24}$ in the male and female subgroups of the nitisinone group of the SONIA 2. (p values indicated for comparison between female pre-nitisinone [n = 24], male pre-nitisinone [n = 45], female nitisinone [n = 120], male pre-nitisinone [n = 225], sampling points where statistical significance was achieved) (data shown as boxplots with median) (statistical significance p expressed *<0.05, **<0.01, and ***<0.001 respectively).

Figure S6. Changes in sHGA/sTYR & $uHGA_{24}/uTYR_{24}$, sHPPA/sTYR & $uHPPA_{24}/uTYR_{24}$, and sHPPA/sHPLA & $uHPPA_{24}/uHPLA_{24}$ in the male and female subgroups of the nitisinone group of the SONIA 2. (p values indicated for comparison between female pre-nitisinone [n = 24], male pre-nitisinone [n = 45], female nitisinone [n = 120], male pre-nitisinone [n = 225], sampling points where statistical significance was achieved) (data shown as boxplots with median) (statistical significance p expressed *<0.05, **<0.01, and ***<0.001 respectively).

Figure S7. The phenylalanine/tyrosine pathway. The PHE/TYR metabolic pathway highlights the site of the enzyme defect observed in AKU and the site of action of nitisinone, a reversible competitive inhibitor of 4-hydroxyphenylpyruvate dioxygenase. The pathway also illustrates the dynamic relationships between HPPA, TYR and HPLA, key interactions after introduction of nitisinone.

Table S1.

Published cases of corneal keratopathy in AKU nitisinone use (REF 18)						
Patient no.	Sex	Age at onset (years)	Dose nitisinone (mg)	Onset keratopathy (months) ¥	Slit-lamp confirmation	sTYR µmol/L≠
NIH Trial						
1	M	48	2	1.5	Y	600
NAC						
2	M	25	2	36, B	Y	964
3	M	21	2	1·5, U	Y	941
4	M	55	2	3, B	N*	NA*
SONIA 2						
5	F	56	10	3, B	Y	1022
6	F	69	10	14, U	N*	1191
7	M	50	10	12, U	Y	1236
8	M	39	10	6, B	Y	1118
9	M	54	10	1, U	Y	816
10	M	44	10	13, U	Y	1036
11	M	29	10	36, B	Y	1149
12	M	41	10	30, U	Y	976
13	M	51	10	24, U	Y	934
14	M	43	10	6, U	Y	609
≠ Last serum tyrosine value before onset of keratopathy or withdrawal of nitisinone; serum tyrosine not always measured at diagnosis of keratopathy<br>¥ U – unilateral; B – bilateral; following nitisinone in months						

Table S2. Dates of start and end of 10mg and 2mg in patients developing keratopathy				
Patient No.	First dose (10mg)	Last dose 10mg	First dose 2mg	Last dose 2mg
1	6/2014	6/2015	8/2015	10/2016
2	9/2014	12/2015	2/2016	9/2018
3	11/2014	11/2015	2/2016	11/2017
4	11/2014	1/2016	-	-
5	2/2015	4/2015	6/2015	12/2018
6	2/2015	3/2016	6/2016	4/2017
7	6/2014	5/2017	7/2017	5/2018
8	9/2014	4/2017	7/2017	11/2018
9	10/2014	10/2016	-	-
10	5/2014	5/2015	7/2015	1/2018
Cells with no dates represent withdrawal from study				

Figure S1.

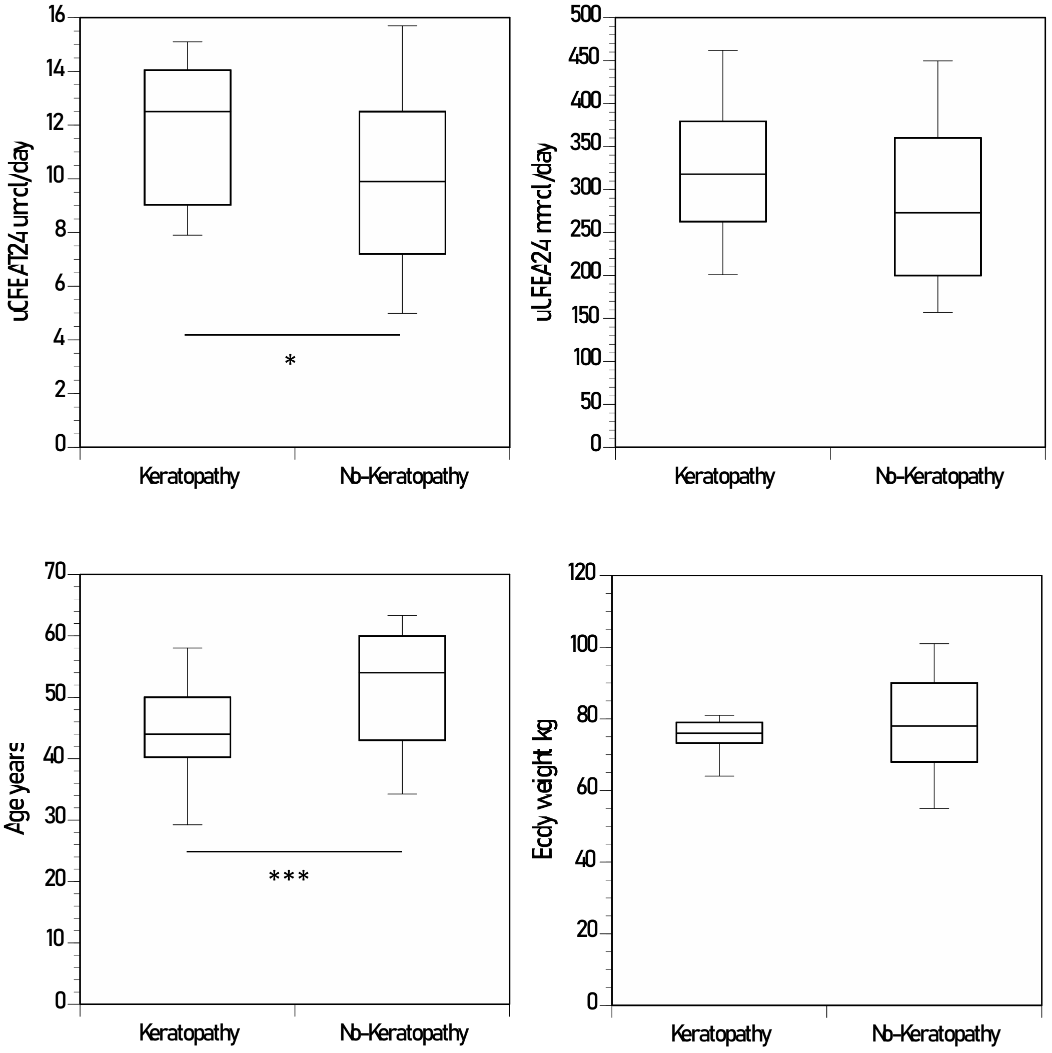

uCFEAT24 umcl/day
Keratopathy
Nb-Keratopathy
*
uLFEA24 mmcl/day
Keratopathy
Nb-Keratopathy
Age years
Keratopathy
Nb-Keratopathy
***
Ecdy weight kg
Keratopathy
Nb-Keratopathy

Figure S2.

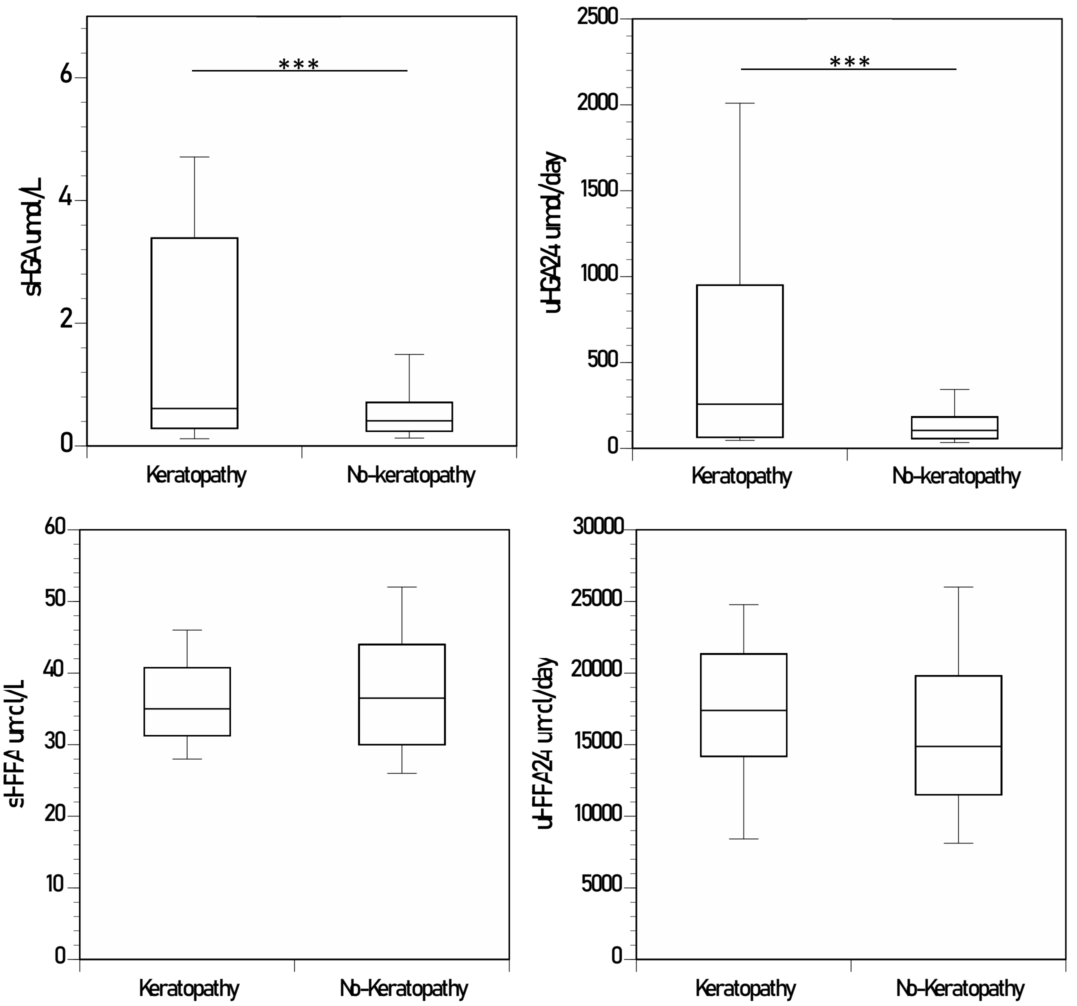

***
sHGA umol/L
Keratopathy
No-keratopathy
***
uHGA24 umol/day
Keratopathy
No-keratopathy
sHFFA umol/L
Keratopathy
No-Keratopathy
uHFFA24 umol/L
Keratopathy
No-Keratopathy

Figure S3.

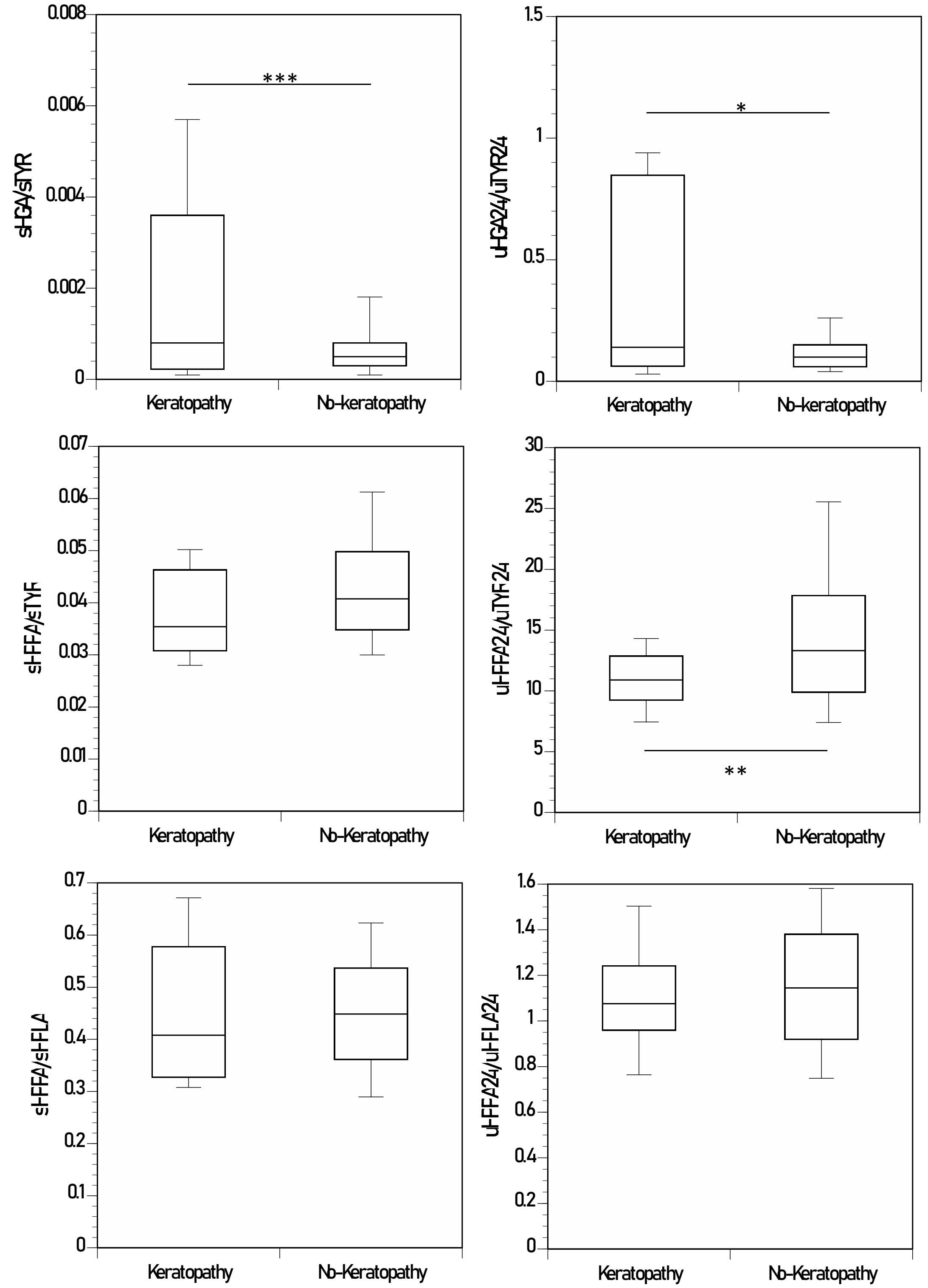

sHGA/sTYR
0.008
0.006
0.004
0.002
0
***
Keratopathy
No-keratopathy
uHGA24/uTYR24
1.5
1
0.5
0
*
Keratopathy
No-keratopathy
sHFFA/sTYR
0.07
0.06
0.05
0.04
0.03
0.02
0.01
0
Keratopathy
No-Keratopathy
uHFFA24/uTYR24
30
25
20
15
10
5
0
**
Keratopathy
No-Keratopathy
sHFFA/sHFLA
0.7
0.6
0.5
0.4
0.3
0.2
0.1
0
Keratopathy
No-Keratopathy
uHFFA24/uHFLA24
1.6
1.4
1.2
1
0.8
0.6
0.4
0.2
0
Keratopathy
No-Keratopathy

Figure S4.

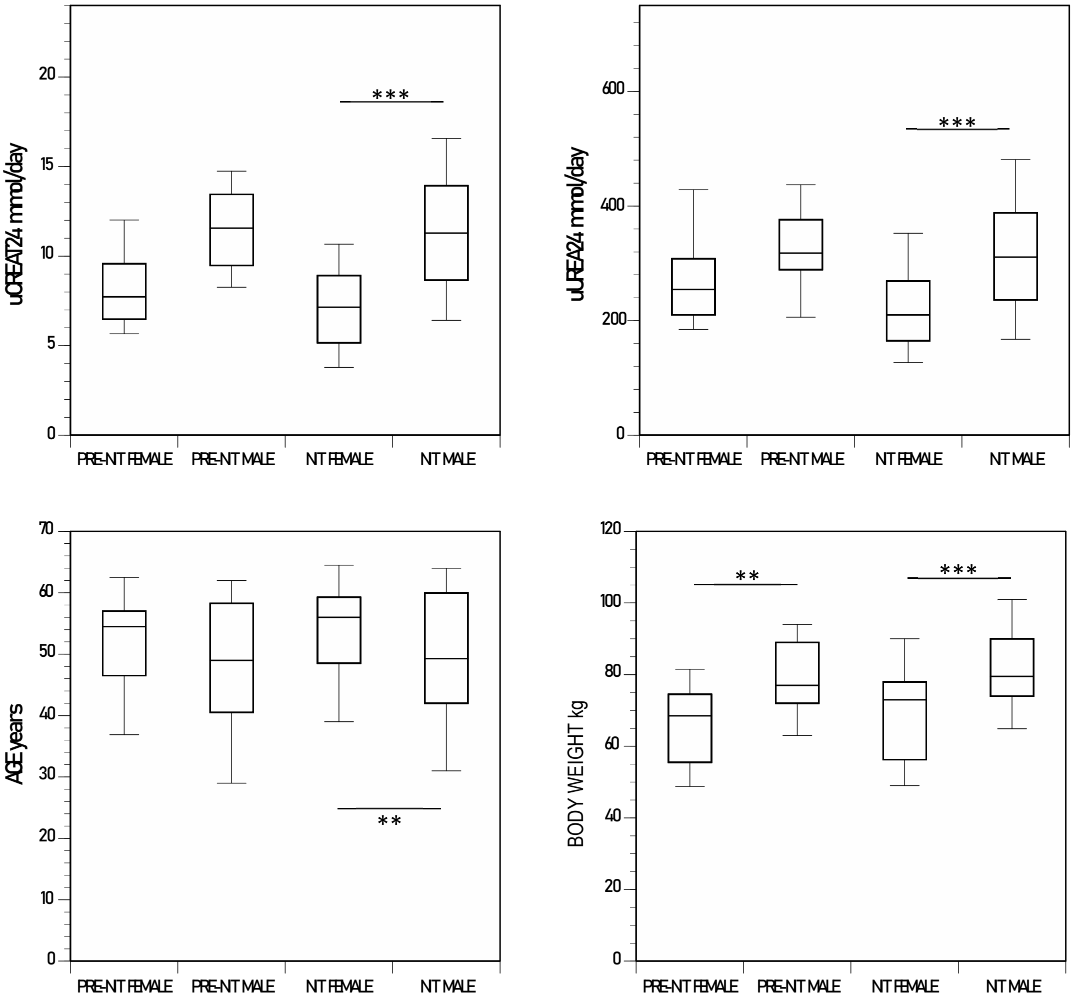
uCREAT24 mmol/day
***
uUREA24 mmol/day
***
AGE years
**
BODY WEIGHT kg
**
***
PRE-NT FEMALE
PRE-NT MALE
NT FEMALE
NT MALE

Figure S5.

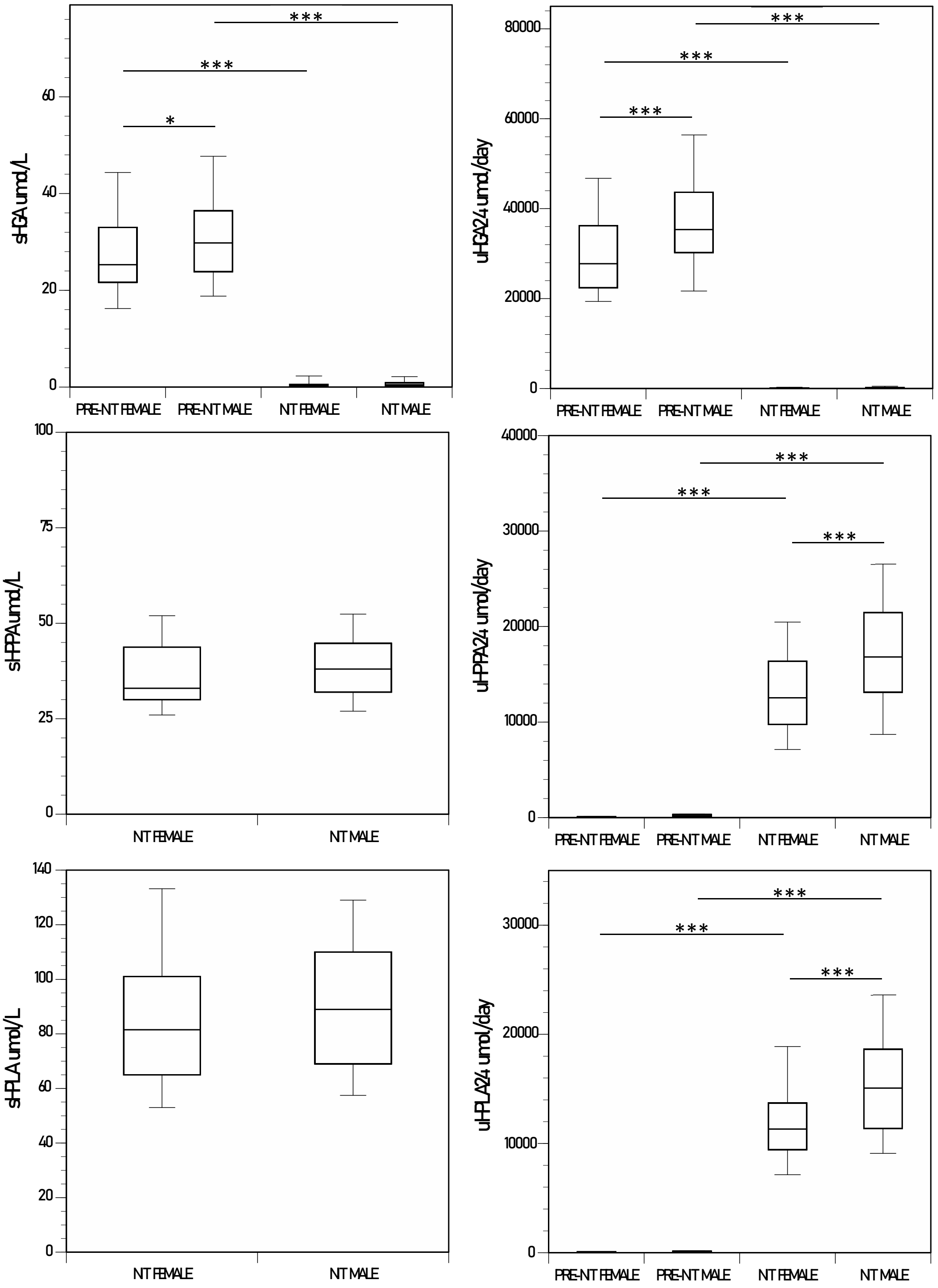
sHGA umol/L
0
20
40
60
PRE-NT FEMALE
PRE-NT MALE
NT FEMALE
NT MALE
*
***
***
uHGA24 umol/day
0
20000
40000
60000
80000
***
***
***
sHPPA umol/L
0
25
50
75
100
uHPPA24 umol/day
0
10000
20000
30000
40000
sHPLA umol/L
0
20
40
60
80
100
120
140
uHPLA24 umol/day
0
10000
20000
30000

Figure S6.

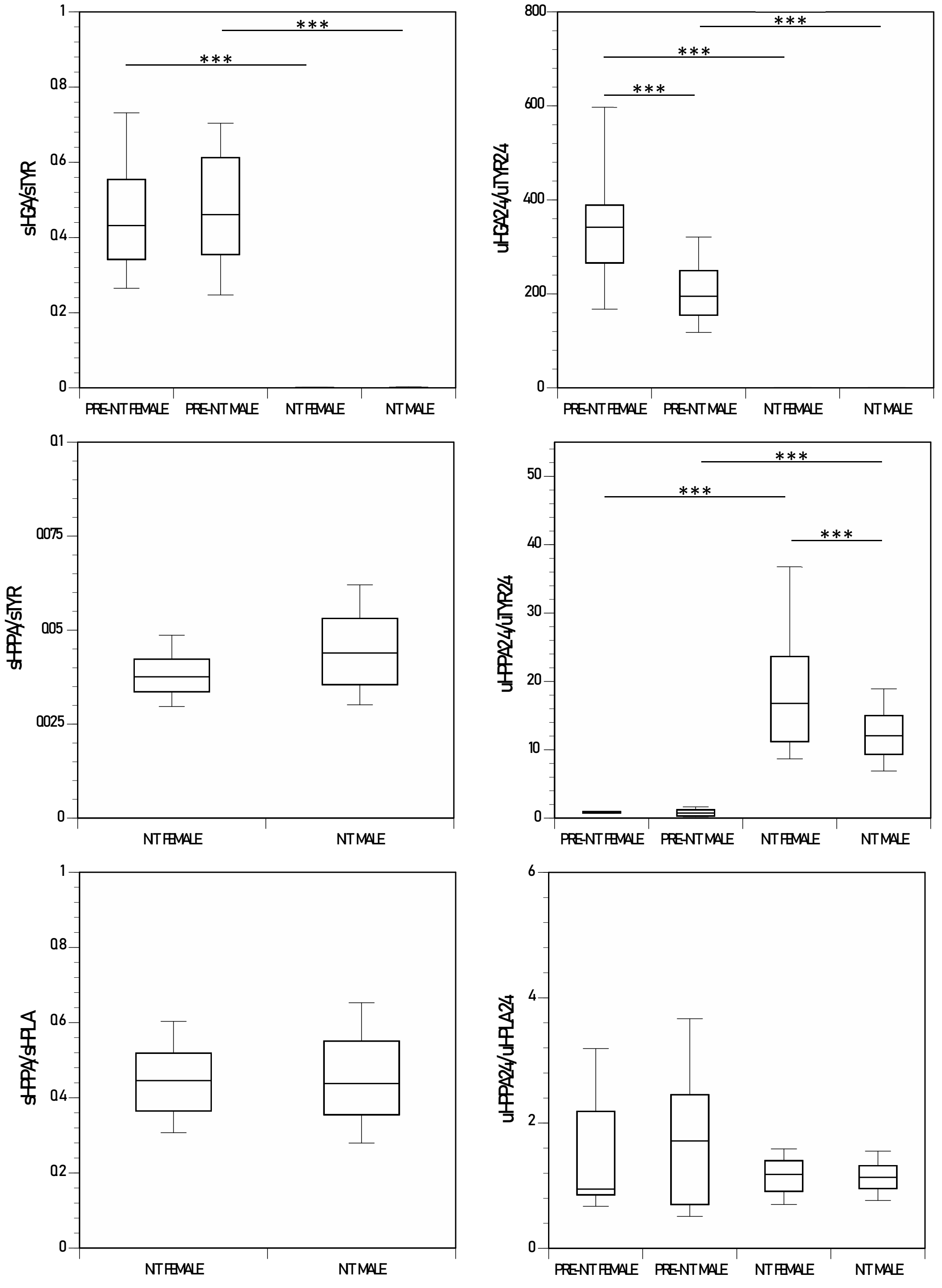
sHGA/sTYR
1
0.8
0.6
0.4
0.2
0
***
***
PRE-NT FEMALE
PRE-NT MALE
NT FEMALE
NT MALE
uHGA24/uTYR24
800
600
400
200
0
***
***
***
PRE-NT FEMALE
PRE-NT MALE
NT FEMALE
NT MALE
sHPPA/sTYR
0.1
0.075
0.05
0.025
0
NT FEMALE
NT MALE
uHPPA24/uTYR24
50
40
30
20
10
0
***
***
***
PRE-NT FEMALE
PRE-NT MALE
NT FEMALE
NT MALE
sHPPA/sHPLA
1
0.8
0.6
0.4
0.2
0
NT FEMALE
NT MALE
uHPPA24/uHPLA24
6
4
2
0
PRE-NT FEMALE
PRE-NT MALE
NT FEMALE
NT MALE

Figure S7.

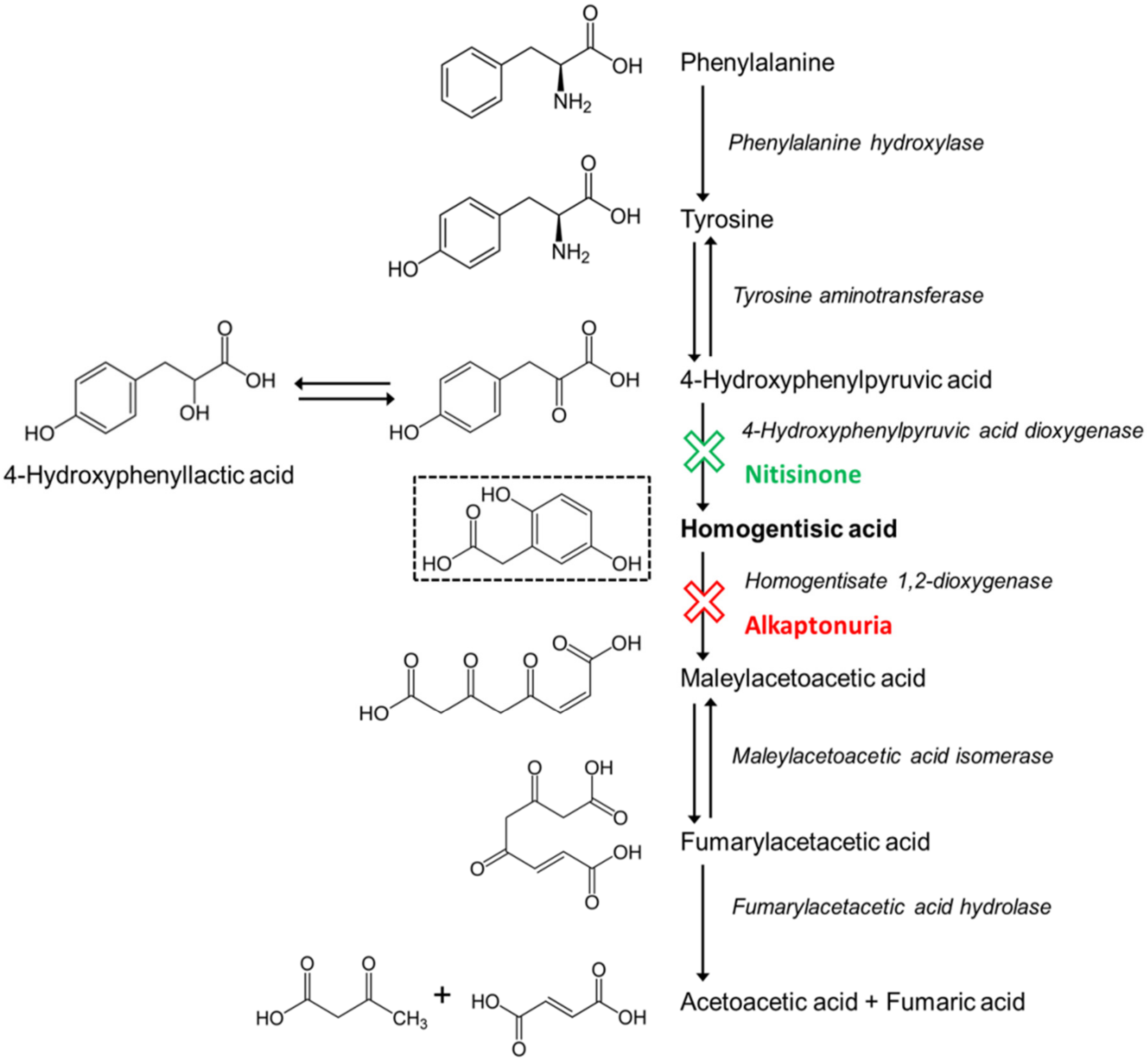

Phenylalanine
Phenylalanine hydroxylase
Tyrosine
Tyrosine aminotransferase
4-Hydroxyphenyllactic acid
4-Hydroxyphenylpyruvic acid
4-Hydroxyphenylpyruvic acid dioxygenase
Nitisinone
Homogentisic acid
Homogentisate 1,2-dioxygenase
Alkaptonuria
Maleylacetoacetic acid
Maleylacetoacetic acid isomerase
Fumarylacetacetic acid
Fumarylacetacetic acid hydrolase
Acetoacetic acid + Fumaric acid
